# A Fluorescent Probe for Glycosaminoglycans Applied to the Detection of Dermatan Sulfate by a Mix-and-Read Assay

**DOI:** 10.3390/molecules22050768

**Published:** 2017-05-09

**Authors:** Melissa Rappold, Ulrich Warttinger, Roland Krämer

**Affiliations:** Inorganic Chemistry Institute, Heidelberg University, Im Neuenheimer Feld 270, 69120 Heidelberg, Germany; melissa.rappold@aci.uni-heidelberg.de (M.R.); ulrich.warttinger@aci.uni-heidelberg.de (U.W.)

**Keywords:** perylene diimide dyes, dermatan sulfate, fluorescent probe, Heparin Red, assay, dermatan sulfate, human plasma

## Abstract

Glycosaminoglycans are complex biomolecules of great biological and medical importance. The quantification of glycosaminoglycans, in particular in complex matrices, is challenging due to their inherent structural heterogeneity. Heparin Red, a polycationic, fluorescent perylene diimide derivative, has recently emerged as a commercial probe for the convenient detection of heparins by a mix-and-read fluorescence assay. The probe also detects glycosaminoglycans with a lower negative charge density than heparin, although with lower sensitivity. We describe here the synthesis and characterization of a structurally related molecular probe with a higher positive charge of +10 (vs. +8 of Heparin Red). The superior performance of this probe is exemplified by the quantification of low dermatan sulfate concentrations in an aqueous matrix (quantification limit 1 ng/mL) and the detection of dermatan sulfate in blood plasma in a clinically relevant concentration range. The potential applications of this probe include monitoring the blood levels of dermatan sulfate after administration as an antithrombotic drug in the absence of heparin and other glycosaminoglycans.

## 1. Introduction

**Scheme 1:**
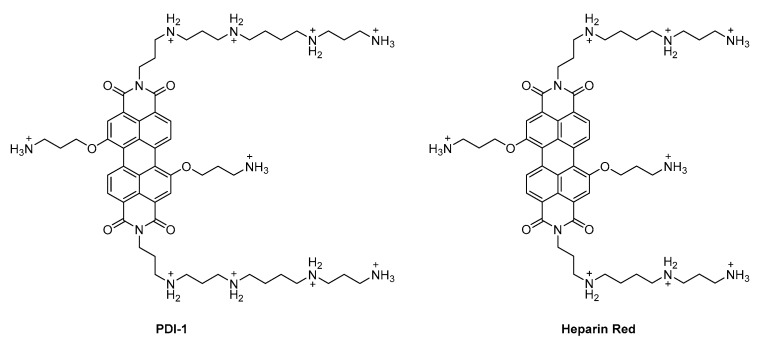
Molecular structures of the probes **PDI-1** and **Heparin Red** in their fully protonated state (charge +10 and +8, respectively).

Glycosaminoglycans are complex biomolecules of great biological and medical importance. They are composed of linear, polydisperse polysaccharides with a highly variable sulfation pattern (with the exception of hyaluronate, which is not sulfated). This structural heterogeneity makes the quantification of glycosaminoglycans challenging. There is a continuous search for more convenient, precise and sensitive quantification methods, in particular for complex biological matrices. Various molecular probes for the direct optical detection of glycosaminoglycans (mainly of heparin, a widely-used antithrombotic drug) have been described [1]. Such probes enable convenient detection by photometry or fluorimetry, although their performance in complex biological matrices is often limited by interference. The fluorescent probe **Heparin Red** has recently emerged as a component of commercially available assays for the quantification of heparins in various matrices [2,3,4]. It is implemented in several drug development projects for the pharmacokinetic monitoring of non-anticoagulant heparins, a promising class of drug candidates [5]. **Heparin Red** is a polyamine modified, red-emissive perylene diimide fluorophore (Scheme 1, right).

**Scheme 2:**
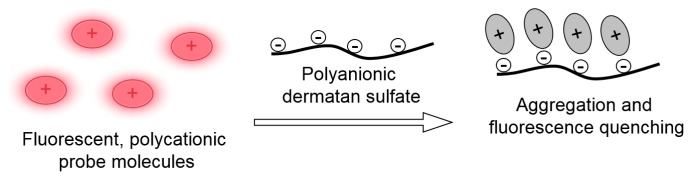
Schematic representation of fluorescence quenching of polycationic probe molecules in the presence of dermatan sulfate due to the formation of non-fluorescent aggregates.

It forms a supramolecular aggregate with glycosaminoglycans, resulting in contact quenching of fluorescence (Scheme 2). The strong binding of the polycationic probe to polyanionic heparin appears to be controlled by both electrostatic and aromatic π-stacking interactions [6]. While electrostatic repulsion between dye molecules prevents spontaneous aggregation in the solution, the charge neutralization by polyanionic heparin favors the formation of π-stacked complexes between the hydrophobic chromophore moieties in the ground state, leading to efficient static quenching. **Heparin Red** has also been applied to the determination of polysaccharides, such as heparan sulfate [7] and fucoidan [8], that have a lower negative charge density than heparin, but the sensitivity in a plasma matrix is significantly lower. An ultrasensitive assay with a quantification limit in the pg/mL range for the highly sulfated polysaccharide dextran sulfate in an aqueous matrix has also been described [9].

**Scheme 3:**
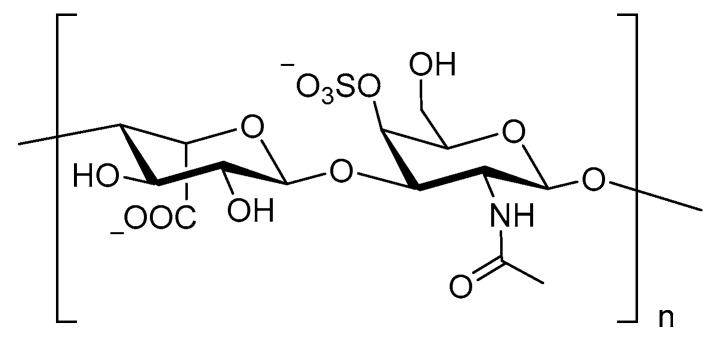
Major iduronic acid-galactosamine repeating disaccharide unit of dermatan sulfate, mono-sulfated form. The sulfation pattern is variable; possible sites of sulfation are galactosamine 4-*O*, 6-*O* and iduronic acid 2-*O*. The disaccharide moiety may be unsulfated, mono- or disulfated.

Dermatan sulfate is a linear, polydisperse sulfated polysaccharide belonging to the glycosamino-glycan family. It is built of a major repeating disaccharide unit consisting of iduronic acid and galactosamine (Scheme 3). The galactosamine may be 4-*O* or 6-*O*-sulfated and the iduronic acid is 2-*O*-sulfated. Typically, the average degree of sulfation is around one per disaccharide. Dermatan sulfate is the predominant glycosaminoglycan expressed in the skin and is released at high concentrations during wound repair. It is also implicated in other biological processes [10], such as development, growth, infection, tumorigenesis and coagulation [11]. Elevated dermatan sulfate levels in plasma or urine have been suggested as a biomarker for certain types of mucopolysaccharidosis [12]. Dermatan sulfate-based drug formulations have been launched for the prevention of venous thromboembolism in Italy (marketed as Mistral) [13] and Portugal (marketed as Venorix). It is also a component of Sulodexide, a more widely-used antithrombotic agent [14]. Dermatan sulfate is produced by extraction from animal tissues, including a complex multistep purification process. The structural heterogeneity makes the direct quantification of dermatan sulfate in complex matrices such as human plasma challenging. Disaccharide analysis [15] provides both quantification and valuable information on dermatan sulfate structure, but involves tedious multistep protocols with sample pretreatment. In clinical settings, dermatan sulfate blood levels are indirectly monitored by coagulation assays [16].

We describe here the direct determination of dermatan sulfate in aqueous and plasma matrices by a new fluorescent probe, **PDI-1** (Scheme 1). The latter is structurally related to **Heparin Red** but has a higher positive molecular charge (+10 vs. +8 for Heparin Red). The enhanced performance of this probe is demonstrated by the determination of dermatan sulfate at low concentrations and in a competitive human plasma matrix.

## 2. Results and Discussion

### 2.1. Synthesis and Properties of the Polyamine-Functionalized Perylene Diimide Probe ***PDI-1***

**Scheme 4:**
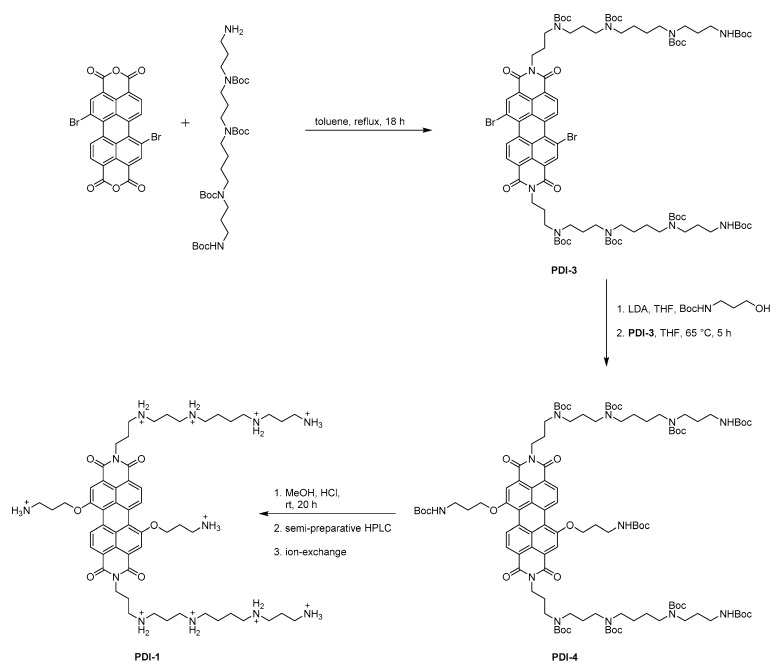
Synthesis of probe **PDI-1**, chloride counterions not shown.

The synthesis of **PDI-1** is described by Scheme 4. A tetra-Boc-protected form of the pentaamine 1,5,9,14,18-pentaza-octadecane was prepared based on a procedure described in the literature [17], and combined with 1,7-dibromoperylene-3,4:9,10-tetracarboxylic acid dianhydride [18] in refluxing toluene to the perylene diimide derivative **PDI-3**. The latter was reacted further with 3-(*N*-Boc)-aminopropanol in tetrahydrofuran, using the base lithium diisopropylamide for the deprotonation of the OH-group; nucleophilic aromatic substitution of bromide led to the fully N-protected intermediate **PDI-4**. The deprotection of **PDI-4** in MeOH/HCl, purification by semi-preparative high-performance liquid chromatography (HPLC) using aqueous trifluoroacetic acid-acetonitrile as an eluent and exchange of the trifluoroacetate counterion with chloride by precipitation from acetonitrile gave the target compound [**PDI-1**]Cl_10_. The probe was characterized by ^1^H- and ^13^C-NMR spectroscopy, electrospray mass spectrometry, UV-Vis spectroscopy, fluorimetry and analytical HPLC. The photophysical properties such as visible absorbance and fluorescence spectra (λ_max (Abs)_ = 576 nm, ε (576 nm) = 35,300 M^−1^ cm^−1^), λ_max_ = 615 nm, quantum yield φ_F_ = 0.17) in a buffered aqueous medium (pH 7) of **PDI-1** are very similar to those of **Heparin Red**.

### 2.2. Detection of Dermatan Sulfate in Aqueous Samples

The response of the probes to dermatan sulfate is controlled by the detection medium; while a high proportion of dimethyl sulfoxide (DMSO) suppresses any response [4], the assay becomes sensitive to dermatan sulfate at a high proportion of water. All detections were performed with spiked samples using commercially available dermatan sulfate sodium salt from porcine intestinal mucosa. A sulfation degree of 1.1 per disaccharide was calculated from the analytical data given in the certificate of the provider (S elemental analysis and water content), and refers to the repeating disaccharide unit given in Scheme 3. The resulting negative charge density per monosaccharide is −1.05, significantly lower in comparison to heparin (−1.7).

**Figure 1:**
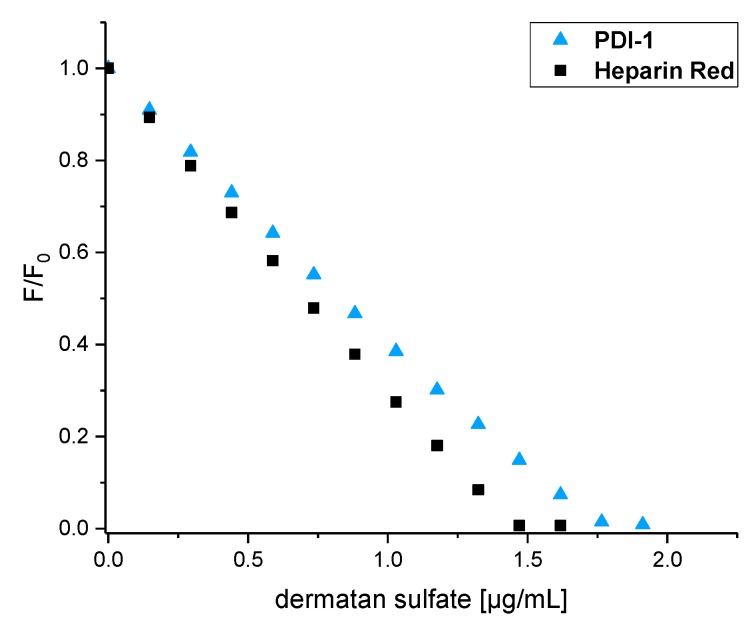
Normalized fluorescence response (F/F_0_) of probes **PDI-1** and **Heparin Red** to dermatan sulfate. Excitation at 550 nm, fluorescence emission recorded at 610 nm. A solution of 1 µM probe in 10 mM MOPS pH 7 was placed into a polymethyl methacrylate (PMMA) cuvette and “titrated” with 10 µL aliquots of a 25 µg/mL dermatan sulfate solution.

Figure 1 shows the titration of 1 µM solutions of the fluorescent probes **PDI-1** and **Heparin Red**, respectively, with dermatan sulfate in an aqueous buffer. A linear decrease of fluorescence intensity with increasing dermatan sulfate concentration was observed. To reach the endpoint of the titration, 19% more dermatan sulfate was needed in case of **PDI-1**. This is in line with the formation of charge neutral, fluorescence-quenched aggregates [6]. Since the charge of **PDI-1** in its fully protonated state is +10 but the fully protonated state of **Heparin Red** only +8, more dermatan sulfate is required for neutralization of the former. The experimental titration endpoints of 1.6 µg/mL for **Heparin Red** and 1.9 µg/mL for **PDI-1** were somewhat lower than the theoretical values of 1.7 and 2.2 µg/mL, respectively, due to the formation of charge-neutral aggregates. The probes may not be fully protonated in the aggregates under the specified conditions, since the proximity of the dye molecules (Scheme 1) could lower the p*K*_a_ value of the ammonium groups.

**Figure 2:**
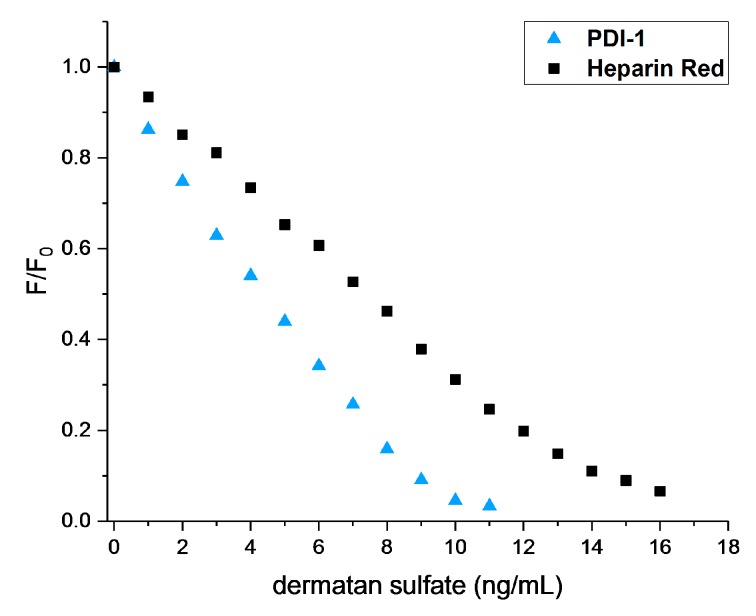
Normalized fluorescence response (F/F_0_) of **PDI-1** and **Heparin Red** to dermatan sulfate. Excitation at 570 nm, fluorescence emission recorded at 610 nm. 2.5 mL water and 0.5 mL probe solution (6 nM in DMSO/30 mM HCl) were mixed in a polystyrene cuvette and the mixture was “titrated” with 5 µL aliquots of a 500 ng/mL dermatan sulfate solution. The indicated dermatan sulfate concentration does not correspond to the actual concentration in the reaction mixture (3 mL) but is related to the 2.5 mL aqueous portion.

We have recently described [9] a modified **Heparin Red** assay that enables the very sensitive quantification in the pg/mL range of the sulfated polysaccharide dextran sulfate in an aqueous matrix. The same protocol (see Materials and Methods for details) enables the highly sensitive detection of dermatan sulfate. Figure 2 compares the titration of a 1 nM solution of **PDI-1** and **Heparin Red**, respectively, with dermatan sulfate. Interestingly, less dermatan sulfate is required for the quenching of probe **PDI-1** under these conditions. The dermatan sulfate/**PDI-1** ratio at the titration endpoint is higher compared with the titration in Figure 1. Apparently, excess dermatan sulfate is needed to shift the equilibrium towards the aggregate at a low concentration of the components. This can only partly be interpreted with a protonation of the carboxylate groups of dermatan sulfate under the assay conditions (5 mM HCl), which may lead to a lower charge density of −0.5 per monosaccharide. Interestingly, even more dermatan sulfate is required for the quenching of **Heparin Red**. This, in contrast to the results in Figure 1, indicates a significantly higher stability of the **PDI-1**-dermatan sulfate aggregates.

**Figure 3:**
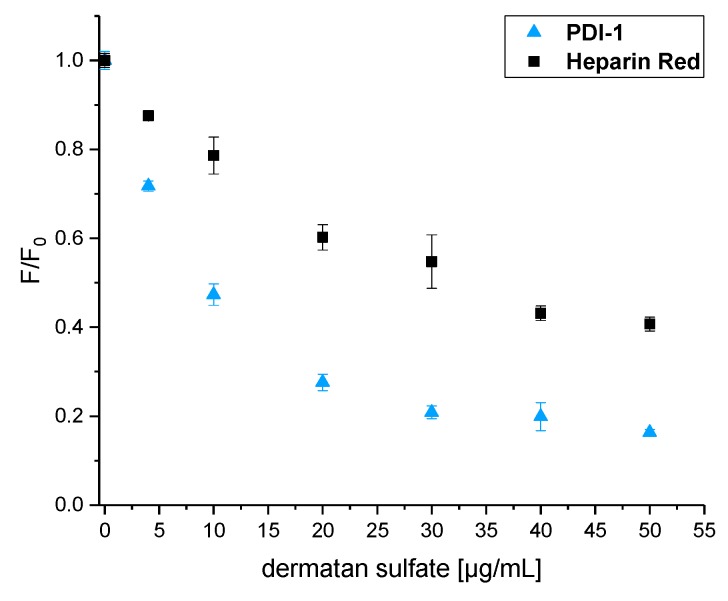
Normalized dose response (F/F_0_) curves of **PDI-1** and **Heparin Red** to dermatan sulfate in human plasma. Spiked pooled normal plasma samples. Manually performed microplate assay was conducted, following the protocol of the supplier for the Heparin Red Kit (using 100-µM solutions of either **Heparin Red** or **PDI-1**). Excitation at 570 nm, fluorescence emission at 605 nm. Averages of duplicate determinations; error bars show standard deviation.

Pooled normal plasma was spiked with dermatan sulfate and the standard protocol for heparin detection by the **Heparin Red** Kit was applied (Figure 3). Obviously, **PDI-1** responded more strongly than **Heparin Red** to the analyte, with about 50% fluorescence quenching at 10 µg/mL. At higher dermatan sulfate levels, the response curve flattens, possibly due to the partial, tight association of the probe with specific plasma components. Apparently, **PDI-1** forms stronger complexes with dermatan sulfate than **Heparin Red** in the human plasma matrix, and detects dermatan sulfate in the clinically relevant concentration range of 1–10 µg/mL. Like **Heparin Red**, **PDI-1** is not selective for dermatan sulfate but also responds strongly to heparin (data not shown), and interference by other glycosaminoglycans that have a similar or higher sulfation degree than dermatan sulfate is expected. A potential application of this probe is the monitoring of blood levels of dermatan sulfate after administration as an antithrombotic drug. Endogenous plasma levels of glycosaminoglycans are too low for significant interference. Only chondroitin sulfate may reach low µg/mL levels but, is present in an undersulfated form [19,20] that does not respond to **Heparin Red** in a plasma matrix.

## 3. Materials and Methods

### 3.1. Chemicals and Reagents

Chemicals used for the synthesis were obtained from Sigma-Aldrich (Taufkirchen, Germany), Merck (Darmstadt, Germany), Acros (Nidderau, Germany), Apollo Scientific (Stockport, UK) or from the central warehouse of Heidelberg University in 95% purity or higher and were used without further purification.

Thin layer chromatography (TLC) was performed on POLYGRAM SIL G/UV_254_ plates from Macherey-Nagel (Düren, Germany) and visualized by UV light. For flash chromatography, silica gel (40–63 µm, 60 Å pore size) from Macherey-Nagel (Düren, Germany) was used.

#### 3.1.1. **Heparin Red** Kit

The **Heparin Red** Kit was a gift from Redprobes UG (Münster, Germany) [21]. Kit components included: **Heparin Red** solution (used for measurements shown in Figure 2), Product No. HR001, Lot 001-003, and Enhancer Solution, Product No. ES001, Lot 005. For the measurements shown in Figure 1 and Figure 3, a solution of **Heparin Red**, synthesized as described in [22], was used.

#### 3.1.2. Dermatan Sulfate

Dermatan sulfate sodium from porcine mucosa, product number C3788 (referred to as chondroitin sulfate B), batch SLBM9912V, was purchased from Sigma-Aldrich. According to the certificate of analysis of the provider, the batch contained 9% water, 6.5% S and 8.6% Na. Identity was confirmed by an ^1^H-NMR spectrum in D_2_O (see Supplementary Materials) and comparison with literature-reported spectra [23]. The lack of signals at >5 ppm indicates the absence of heparin and heparan sulfate. The *N*-Acetyl resonance and spiking experiments suggested that the sample may have contained minor impurities of chondroitin sulfate A and/or C [24].

#### 3.1.3. Plasma

Pooled human normal plasma, applied as a matrix for the detections shown in Figure 3, was obtained from PrecisionBioLogic (Dartmouth, NS, Canada): Lot A1161, CRYOcheck. A 100 µg/mL stock solution of dermatan sulfate in plasma was prepared by mixing a 1 mg/mL aqueous dermatan sulfate solution with plasma in a volume ratio 1:9, followed by further dilution with plasma to the desired concentration. All samples were stored at −20 °C for at least one day and up to several months, and were thawed at room temperature and vortexed before use.

#### 3.1.4. Other

All aqueous solutions were prepared with HPLC grade water purchased from VWR (Bruchsal, Germany), product No. 23595.328. DMSO, product number 34869, Lot # STBF6384V, and hydrochloric acid (1.0 M), product number 35328, Lot # SZBF2050V, were purchased from Sigma-Aldrich.

### 3.2. Instrumentation

#### 3.2.1. Analytics

Nuclear magnetic resonance spectra were recorded on a Bruker Avance III (600 MHz) spectrometer (Bruker Corporation, Billercia, MA, USA). Chemical shifts δ are given in ppm and coupling constants *J* in Hz. All spectra were recorded at 295 K and calibrated using the residual ^1^H- or ^13^C-signals of the deuterated solvents [25]. The following abbreviations were used to describe the multiplicities of the signals: s (singlet), d (doublet), m (multiplet), b (broad). Signals were assigned using COSY, HSQC and HMBC spectra. Mass spectra were recorded on a Bruker ApexQe Hybrid 9.4 FT-ICR (Bruker Corporation, Billercia, MA, USA). Semi-preparative and analytical HPLCs were performed on a Shimadzu HPLC system at 20 °C using a SPD-10Avp detector (semi-preparative) or a SPD-M20A Photodiode Array Detector (analytical), respectively. Water with 0.1% trifluoracetic acid and acetonitrile were used as eluents and C18 columns were used as the stationary phase. UV/Vis spectra were recorded on a Varian Cary 100 Bio UV/Vis and fluorescence-spectra on a Varian Cary Eclipse spectrometer (Varian Inc., Palo Alto, CA, USA) using 4.5 mL PMMA-cuvettes purchased from Sigma-Aldrich.

#### 3.2.2. Fluorescence Measurements

Fluorescence in microplates was measured with the reader Biotek Synergy Mx (Biotek Instruments, Winooski, VT, USA), with an excitation at 570 nm, emission recorded at 605 nm, spectral band width 13.5–17 nm, gain 90–110, read height of 8 mm. Fluorescence in cuvettes was measured with either (Figure 1) Trilogy Laboratory Fluorimeter (Trilogy Modul RWT/PE, GUI Selection Green, excitation at 550 nm, emission recorded at 610 nm) or (Figure 2) the benchtop fluorimeter FluoroLog-3 (Horiba Scientific, Kyoto, Japan), with an excitation at 570 nm, emission recorded at 590–650 nm, integration time 0.4 s, average of 3 scans, and spectral band width Ex/Em 7 nm.

#### 3.2.3. Microplates, Cuvettes, Pipettes

For fluorescence measurements, 96-well microplates, polystyrene, Item No. 655076, were pur­chased from Greiner Bio-One GmbH (Frickenhausen, Germany). Disposable PMMA cuvettes were used for absorbance or fluorescence measurements in aqueous medium. Disposable polystyrene fluorescence cuvettes were used for measurements in water/DMSO medium. Transferpette 0.5–10 µL, Transferpette-8 20–200 µL and Transferpette-12 20–200 µL, purchased from Brand GmbH (Wertheim, Germany). Rainin Pipettes 100–1000 µL, 20–200 µL, and 2–20 µL purchased from Mettler Toledo (Columbus, OH, USA).

### 3.3. Synthesis

To improve comprehensibility, simplified names were used for some synthesized compounds rather than using exact IUPAC names. 1,7-dibromoperylene-3,4:9,10-tetracarboxylic acid dianhydride [18], *N*^1^,*N*^4^,*N*^9^,*N*^13^-Tetra-*tert*-butyloxycarbonyl-1,16-diamino-4,9,13-triazahexadecane [17] and *N*,*N*’-Bis-(1-amino-4,9-diiazadodecyl)-1,7-di-(1-amino-3-hydroxypropyl)-perylene-3,4:9,10-tetracarboxydiimide (**Heparin Red**) [2,26] were prepared according to literature-reported methods which were slightly modified [22].

#### 3.3.1. Synthesis of *N*,*N*’-Bis-(*N*^1^,*N*^4^,*N*^9^*,N*^13^-tetra-*tert*-butyloxycarbonyl-1-amino-4,9,13-triazahexa­decyl)-1,7-dibromperylene-3,4:9,10-tetracarboxylic acid bisimide **PDI-3**

*N*^1^,*N*^4^,*N*^9^,*N*^13^-Tetra-*tert*-butyloxycarbonyl-1,16-diamino-4,9,13-triazahexadecane (600 mg, 909 µmol) was dissolved in anhydrous toluene (20 mL) prior to the addition of 1,7-dibromoperylene-3,4:9,10-tetracarboxylic acid dianhydride (227 mg, 413 µmol). The reaction mixture was stirred under reflux for 18 h. Insoluble residues were removed by filtration. The filtrate was evaporated to dryness and the crude product was purified by column chromatography (SiO_2_, CH_2_Cl_2_:MeOH—98:2). Compound **PDI-3** was isolated as a red powder (302 mg, 165 µmol, 40%). *R*_f_ (CH_2_Cl_2_:MeOH—95:5) 0.50; ^1^H-NMR (600.13 MHz, CDCl_3_): δ (ppm) = 9.50 (d, ^3^*J*_H-H_ = 8.17 Hz, 2H, perylene-H), 8.92 (s, 2H, perylene-H), 8.71 (d, ^3^*J*_H-H_ = 8.17 Hz, 2H, perylene-H), 4.17–4.27 (m, 4H, CH_2_), 3.31–3.40 (m, 4H, CH_2_), 3.07–3.26 (m, 24H, CH_2_), 1.95–2.03 (m, 4H, CH_2_), 1.73–1.83 (m, 4H, CH_2_), 1.62–1.69 (m, 4H, CH_2_), 1.50–1.53 (m, 8H, CH_2_), 1.41–1.46 (m, 72H, Boc-H); ^13^C{^1^H}-NMR (150.91 MHz, CDCl_3_): δ (ppm) = 138.0, 130.2, 128.6, 46.6, 44.8, 43.8, 38.6, 37.5, 28.5, 28.4, 27.6, 27.0, 25.8; MS (HR-ESI^+^): *m*/*z* = 1853.7890 [M + Na]^+^, calculated for C_90_H_132_^79^Br_2_N_10_NaO_20_^+^: 1853.7878, *m*/*z* = 1831.8037 [M + H]^+^, calculated for C_90_H_133_^79^Br_2_N_10_O_20_^+^: 1831.8059.

#### 3.3.2. Synthesis of *N*,*N*’-Bis-(1-amino-4,9,13-triazahexadecyl)-1,7-di-(1-amino-3-hydroxypropyl)-perylene-3,4:9,10-tetracarboxydiimide **PDI-1**

Compound **PDI-3** (20.0 mg, 19.9 µmol) was dissolved in anhydrous tetrahydrofurane (THF, 2 mL). In another flask, 3-(*N*-(*tert*-butyloxycarbonyl)-amino)1-propanol (191 mg, 1.09 mmol) was dissolved in anhydrous THF (2 mL) prior to the addition of lithium diisopropylamide (2.0 M solution in THF, 500 µL). This mixture was then added to the first flask and stirred for 5 h at 65 °C. The cooled reaction mixture was diluted with an excess of ethyl acetate. The organic layer was washed with brine and water, dried with anhydrous Na_2_SO_4_, and concentrated under reduced pressure. The crude product (**PDI-4**) was purified by column chromatography (SiO_2_, CH_2_Cl_2_:MeOH—98:2) to afford a violet solid, which was used without further purification. *R*_f_ (CH_2_Cl_2_:MeOH—95:5) 0.28; MS (HR-ESI^+^): *m*/*z* = 2045.1841 [M + Na]^+^ calculated for C_106_H_164_N_12_NaO_26_^+^: 2045.1850, *m*/*z* = 1034.0868 [M + 2Na]^2+^ calculated for C_106_H_164_N_12_Na_2_O_26_^2+^: 1034.0849.

The crude product (**PDI-4**) was dissolved in methanol (10 mL), and concentrated HCl (1 mL) was added to remove the Boc-protecting groups. This reaction mixture was stirred for 20 h at room temperature. The solvent was removed under reduced pressure. Purification by semi-preparative HPLC (Macherey-Nagel C18 (250 mm × 10 mm), H_2_O with 0.1% trifluoroacetic acid (TFA)/MeCN, 4 mL/min, 0–5 min—0% MeCN, 15 min—12% MeCN, 30–35 min—24% MeCN, 55 min—90% MeCN) and subsequent lyophilization yielded the TFA salt of **PDI-1** (1.53 mg, 1.50 µmol, 8%) as a violet solid.

The trifluoroacetate counterions were exchanged by dissolving the product in 0.6 M HCl followed by precipitation from acetonitrile. After washing with acetonitrile, re-dissolution in water and subsequent lyophilization, the target compound [**PDI-1**]Cl_10_ was ready to use. ^1^H-NMR (600.13 MHz, MeOD): δ (ppm) = 9.48–9.58 (m, 2H, perylene-H), 8.63 (d, ^3^*J*_H-H_ = 8.28 Hz, 2H, perylene-H), 8.45 (bs, 2H, perylene-H), 4.58–4.71 (m, 4H, CH_2_), 4.32–4.40 (m, 4H, CH_2_), 3.32–3.35 (m, 4H, CH_2_, superimposed by MeOD), 3.03–3.23 (m, 32H, CH_2_, N–H), 2.47–2.53 (m, 4H, CH_2_), 2.14–2.26 (m, 8H, CH_2_), 2.05–2.11 (m, 4H, CH_2_), 1.79–1.83 (m, 8H, CH_2_); ^13^C{^1^H}-NMR (150.90 MHz, MeOD): δ (ppm) = 130.0, 129.5, 68.8, 48.3, 47.0, 45.7, 46.0, 38.4, 38.0, 37.9, 28.2, 25.9, 25.1, 24.0, 23.9; MS (HR-ESI^+^): *m*/*z* = 1021.6722 [M + H]^+^ calculated for C_56_H_85_N_12_O_6_^+^: 1021.6710 *m*/*z* = 511.3395 [M + 2 H]^2+^, calculated for C_56_H_86_N_12_O_6_^2+^: 511.3391; UV/Vis (H_2_O, 10 mM MOPS, pH 7): λ_max (Abs)_ (ε [M^−1^ cm^−1^]) = 402 nm (5,900), 542 nm (24,100), 576 nm (35,300); Fluorescence (H_2_O, 10 mM MOPS, pH 7): λ_Ex_ = 530 nm, λ_Abs(Em)_ = 615 nm, φ_F_ = 0.17; Analytical HPLC: (Macherey-Nagel C18 (250 mm × 4.6 mm), H_2_O with 0.1% TFA/MeCN, 1 mL/min, 0–5 min—0% MeCN, 15 min—12% MeCN, 30–35 min—24% MeCN, 55 min—90% MeCN) *t*_R_ = 31.6 min.

### 3.4. Assays

#### Dermatan Sulfate Assay in Aqueous and Plasma Matrix

For titrations of probe solutions with dermatan sulfate, the solutions described in the legends of Figure 1 and Figure 2 of **PDI-1** and **Heparin Red**, respectively, were prepared using 100 µM aqueous stock solutions of the probes. Then, 6-nM probe solutions in DMSO/30 mM HCl (Figure 2) were prepared as described in detail in [9] for **Heparin Red**. Dermatan sulfate in spiked plasma samples was detected as described for heparin, following the protocol of the provider of the **Heparin Red** Kit for a 96-well microplate, using either a 100-µM aqueous solution of **Heparin Red** or of **PDI-1**. In brief, the 100-µM solution of **PDI-1** or **Heparin Red** was freshly mixed with Enhancer solution at a volume ratio of 1:90. Subsequently, 20 µL of the dermatan sulfate spiked plasma sample was pipetted into a microplate well, followed by 80 µL of the probe—Enhancer mixture. For sample numbers >10, a 12-channel pipette was used for the addition of the probe—Enhancer solution. The microplate was introduced in the fluorescence reader and mixing was performed using the plate shaking function of the microplate reader (setting “high,” 3 min). Immediately after mixing, fluorescence was recorded within 1 min.

## 4. Conclusions

This contribution describes the synthesis and properties of the new fluorescent probe **PDI-1**, a polyamine-functionalized perylene diimide. **PDI-1** is structurally related to the commercial probe **Heparin Red**, but has in its protonated form a higher molecular charge of +10, compared to +8 for **Heparin Red**. The superior performance of **PDI-1** for sensing of a glycosaminoglycan with low negative charge density is exemplified by the detection of dermatan sulfate at a low concentration or in a competitive blood plasma matrix. A potential application of this probe is the direct monitoring of the antithrombotic drug dermatan sulfate in plasma in the absence of administered heparin and other glycosaminoglycans.

## Supplementary Materials

Supplementary materials are available online.
