# Supplementary material for: A Fluorescent Probe for Glycosaminoglycans Applied to the Detection of Dermatan Sulfate by a Mix-and-Read Assay
Source: Molecules. 2017 May 9;22(5):768. doi: 10.3390/molecules22050768 (PMC6154688; doi:10.3390/molecules22050768)

Supporting Material

^{1}H-NMR (600.13 MHz, MeOD) of **PDI-1**

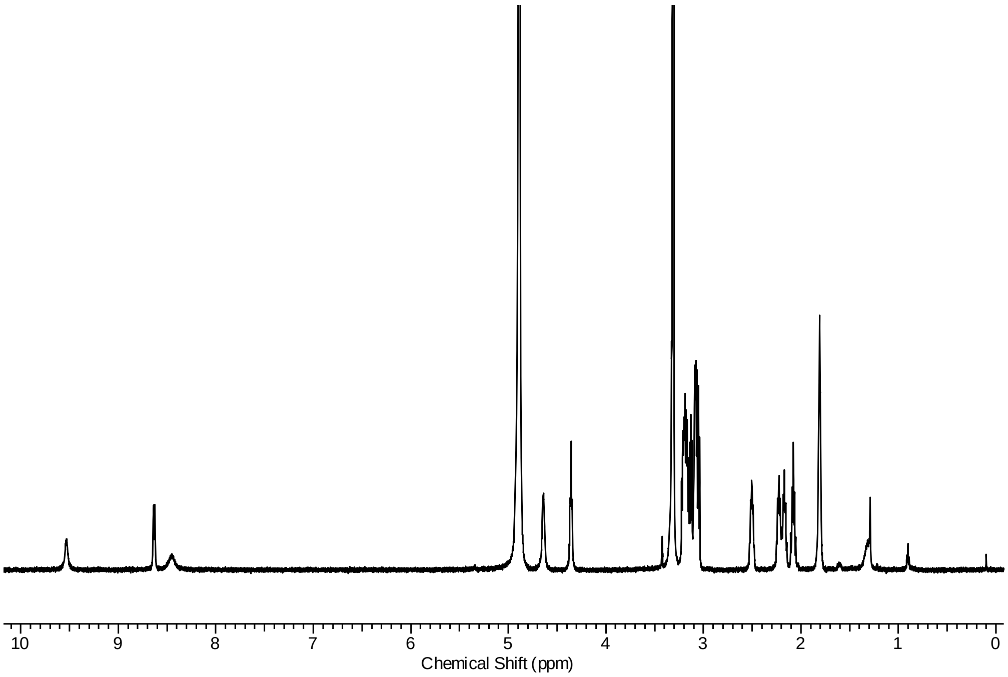

HR-ESI^{+} mass spectrum of **PDI-1**

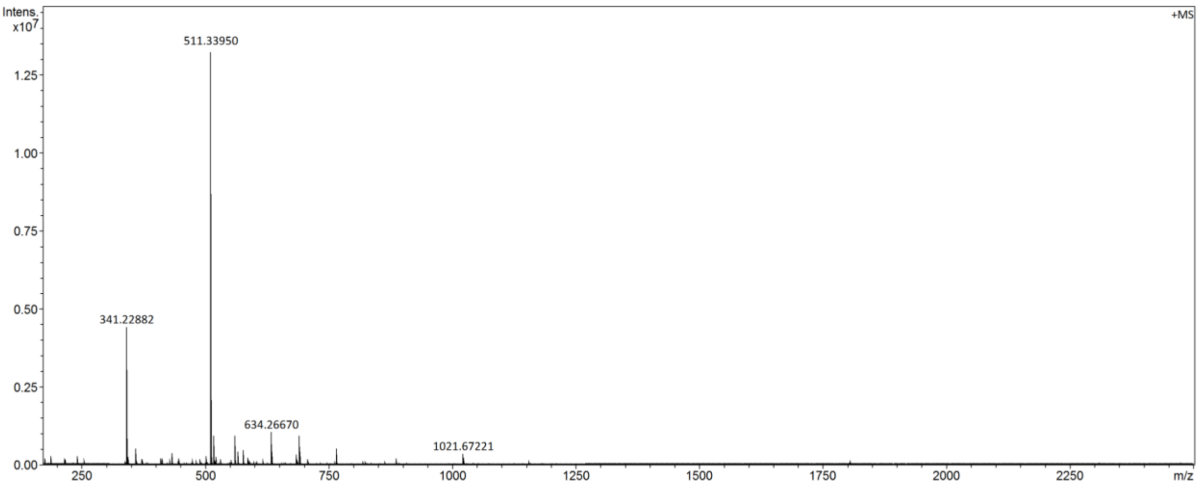

Absorption- and Emissionspectra in H_2O, 10 mM MOPS buffer pH 7 (c(PDI) = 2 µM; λ_{Ex} = 575 nm, U_D = 500 V) and analytical HPLC of **PDI-1**

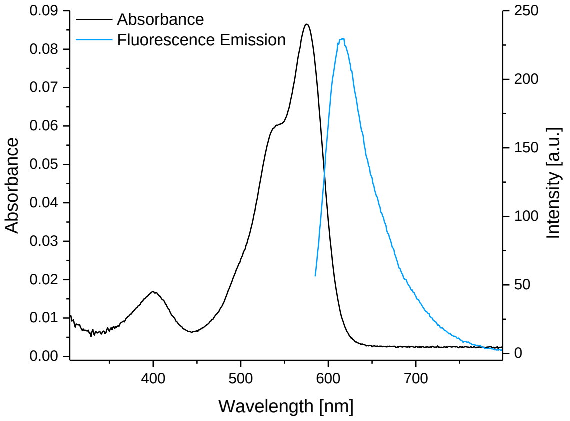

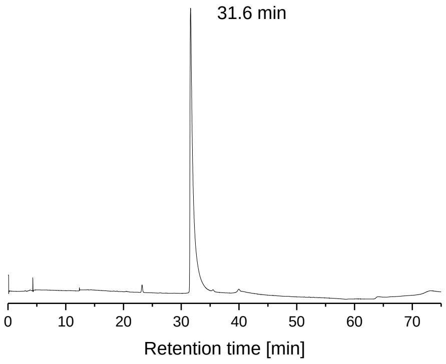

^{1}H-NMR (600.13 MHz, D_2O, c = 6 mg/mL) of commercial dermatan sulfate sample, reference tert-butanol

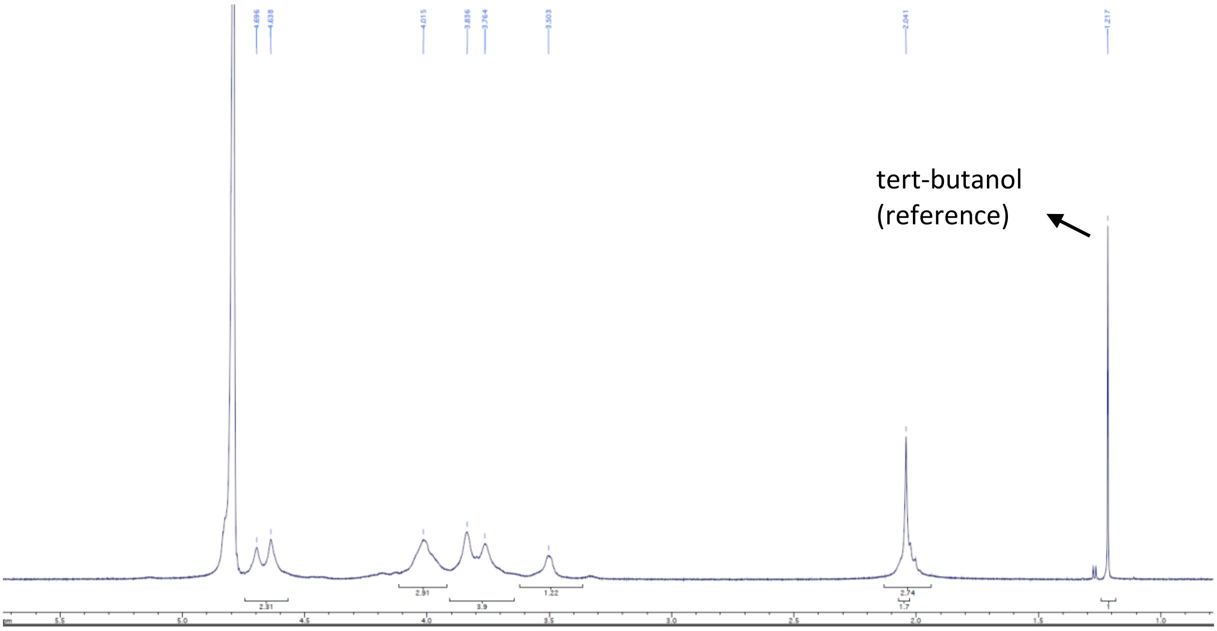

^{1}H-NMR (600.13 MHz, D_2O, c = 7 mg/mL) of commercial dermatan sulfate sample, reference tert-butanol; after spiking with 2.4 mg/mL chondroitin sulfate A/C mixture

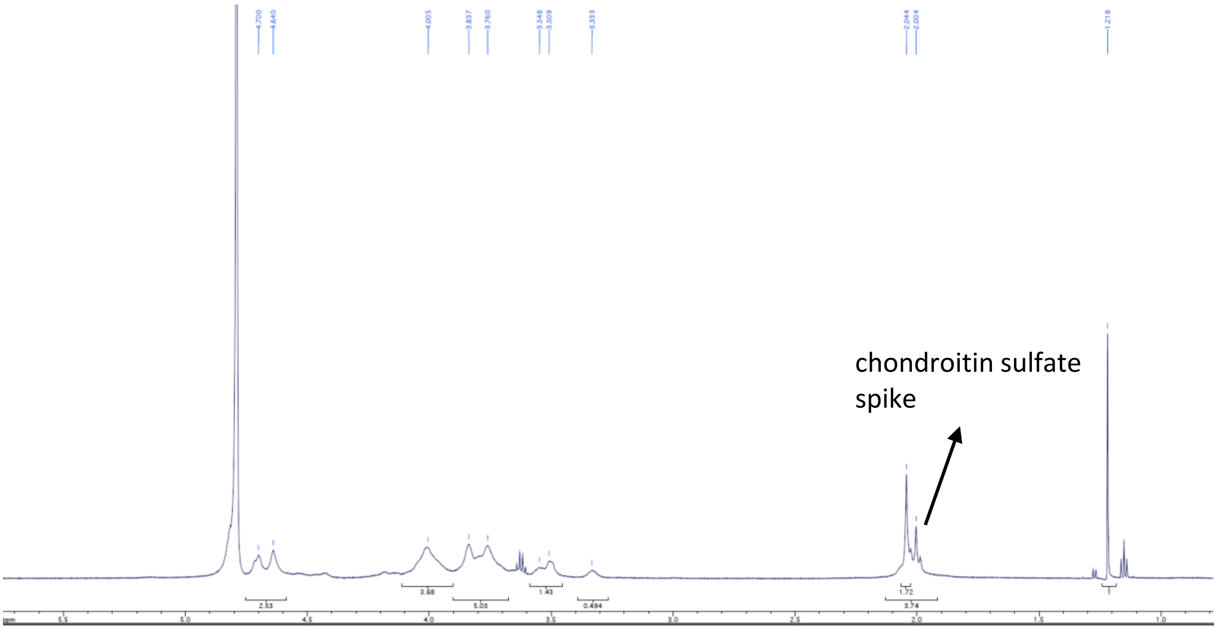

Supplement: Supplementary file 1 [file molecules-22-00768-s001.pdf]
